# Challenging and Dismantling Ageist Attitudes, Beliefs, and Behaviors Through Intergenerational Programs

**DOI:** 10.1093/geroni/igaa057.1552

**Published:** 2020-12-16

**Authors:** Tia Rogers-Jarrell, Brad Meisner

**Affiliations:** 1 York University, Mississauga, Ontario, Canada; 2 York University, North York, Ontario, Canada

## Abstract

Age stereotypes are complex and multifaceted: individuals can demonstrate and embody numerous and varied positive and negative stereotypes. Therefore, solutions to combat age stereotypes must also be complex and multifaceted. Additionally, both social and physical forms of age segregation are common in our society. This causes fewer and fewer opportunities for younger and older people to interact. Intergroup Contact Theory suggests age stereotypes can be reduced through increased intergenerational contact. One way to encourage contact between younger and older populations is through intergenerational programming. However, there is a lack of literature investigating the effects of intergenerational programs on perceptions of aging. The purpose of this paper was to critically review and explore literature on intergenerational programs to understand how they influence age stereotypes and ageist attitudes. The available literature suggests that intergenerational programs involving young children (ages 4-8), adolescents (ages 11-18), or emerging adults (ages 19-26) interacting with older adults (ages 65+) can significantly reduce age stereotypes towards older adults. Additionally, older adults (ages 65+) negative beliefs and attitudes towards younger people (ages 4-26) can also be deconstructed after participation in intergenerational programs. Intergenerational programs act to break down age barriers and promote connections and understandings between generations. These programs challenge the belief that older and younger people should live and participate in spaces that are separate from one another. Providing opportunities for younger and older people to participate in intergenerational programs is one way to promote respectful relationships and enhance the quality of life and health of all generations.

